# A risk nomogram for predicting prolonged intensive care unit stays in patients with chronic obstructive pulmonary disease

**DOI:** 10.3389/fmed.2023.1177786

**Published:** 2023-07-06

**Authors:** Hongtao Cheng, Jieyao Li, Fangxin Wei, Xin Yang, Shiqi Yuan, Xiaxuan Huang, Fuling Zhou, Jun Lyu

**Affiliations:** ^1^School of Nursing, Jinan University, Guangzhou, China; ^2^Intensive Care Unit, The First Affiliated Hospital of Jinan University, Guangzhou, China; ^3^Department of Neurology, The First Affiliated Hospital of Jinan University, Guangzhou, China; ^4^Department of Hematology, Zhongnan Hospital of Wuhan University, Wuhan, China; ^5^Department of Clinical Research, The First Affiliated Hospital of Jinan University, Guangzhou, China; ^6^Guangdong Provincial Key Laboratory of Traditional Chinese Medicine Informatization, Guangzhou, China

**Keywords:** chronic obstructive pulmonary disease, intensive care unit, length of stay, nomograms, prolonged intensive care unit stays

## Abstract

**Background:**

Providing intensive care is increasingly expensive, and the aim of this study was to construct a risk column line graph (nomograms)for prolonged length of stay (LOS) in the intensive care unit (ICU) for patients with chronic obstructive pulmonary disease (COPD).

**Methods:**

This study included 4,940 patients, and the data set was randomly divided into training (*n* = 3,458) and validation (*n* = 1,482) sets at a 7:3 ratio. First, least absolute shrinkage and selection operator (LASSO) regression analysis was used to optimize variable selection by running a tenfold k-cyclic coordinate descent. Second, a prediction model was constructed using multifactorial logistic regression analysis. Third, the model was validated using receiver operating characteristic (ROC) curves, Hosmer-Lemeshow tests, calibration plots, and decision-curve analysis (DCA), and was further internally validated.

**Results:**

This study selected 11 predictors: sepsis, renal replacement therapy, cerebrovascular disease, respiratory failure, ventilator associated pneumonia, norepinephrine, bronchodilators, invasive mechanical ventilation, electrolytes disorders, Glasgow Coma Scale score and body temperature. The models constructed using these 11 predictors indicated good predictive power, with the areas under the ROC curves being 0.826 (95%CI, 0.809–0.842) and 0.827 (95%CI, 0.802–0.853) in the training and validation sets, respectively. The Hosmer-Lemeshow test indicated a strong agreement between the predicted and observed probabilities in the training (χ^2^ = 8.21, *p* = 0.413) and validation (χ^2^ = 0.64, *p* = 0.999) sets. In addition, decision-curve analysis suggested that the model had good clinical validity.

**Conclusion:**

This study has constructed and validated original and dynamic nomograms for prolonged ICU stay in patients with COPD using 11 easily collected parameters. These nomograms can provide useful guidance to medical and nursing practitioners in ICUs and help reduce the disease and economic burdens on patients.

## Introduction

Chronic obstructive pulmonary disease (COPD) is a common progressive disease with persistent respiratory symptoms and airflow limitation caused by the combination of chronic bronchitis and emphysema (1). It is one of the most common chronic diseases worldwide that can lead to prolonged cough, dyspnea, and fatigue, and accounts for a large proportion of intensive care unit (ICU) admissions (2, 3). In the year 2019, a staggering number of 212.3 million cases of COPD were reported worldwide, leading to 3.3 million fatalities and 74.4 million years of disability adjusted life (4). Furthermore, in the same year, COPD ranked as the third most prevalent cause of death (5). Due to its high prevalence, morbidity, and mortality, COPD is a significant area of concern for both medical care and public health (6). Furthermore, research has shown that COPD imposes a considerable economic and social burden on patients and healthcare systems (7, 8). Efforts to prevent and manage COPD are crucial not only for improving patient outcomes but also for reducing the economic and societal impact of the disease.

An ICU is a place where medical professionals apply modern medical theories and high-technology modern medical equipment to provide specialized centralized monitoring, treatment, and care for critically ill patients, and is an integral part of the healthcare system (9, 10). ICUs provide complex and expensive care (11), and account for a significant portion of the financial expense of healthcare in many countries worldwide (12), for example, in the United States, ICU medical costs account for approximately 13% of hospital costs and 4% of national health expenditure (13). Despite the enormous investment in critical care medicine in many countries, ICUs are often underresourced to meet the needs of critically ill patients, especially in less-developed countries (14). Length of stay (LOS) in the ICU is a key indicator of healthcare efficiency and an important indicator of the quality of critical care provided by a hospital (15, 16). Prolonged ICU stays often result in large resource utilization and thus markedly increased healthcare costs (17). A multistate, multihospital analysis found that ICU utilization rates varied widely among patients hospitalized with COPD. It is therefore particularly important to determine the factors that prolong LOS in the ICU for patients with COPD in order to help accelerate ICU bed turnover, reduce patient care and financial burden, and prevent poor prognoses.

The literature (18–22) has suggested that LOS in hospitalized patients with COPD may be influenced by various factors, such as age, smoking history, Charlson Comorbidity Index, comorbidities, malnutrition, mobility, and mechanical ventilation. Some vital-sign parameters such as higher respiratory rate on admission, systolic blood pressure > 140 mmHg, and diastolic blood pressure > 90 mmHg have also been considered as risk factors for LOS (23, 24). However, the factors that influence the length of ICU stay for patients with COPD are not well defined.

Previous nomograms (25) often suffered from small sample sizes, which could limit the generalizability of their results and hinder their applicability in diverse populations of critically ill patients. Moreover, these models relied on predictors that were difficult or time-consuming to collect, making them less practical for real-world clinical application. Additionally, the representation of the entire spectrum of severity among critically ill patients was not always achieved in previous models, potentially affecting their predictive accuracy and clinical usefulness. In this study, we aimed to address these limitations by constructing a novel nomogram for predicting prolonged ICU stays in patients with COPD. We utilized the large and reliable MIMIC-IV (Medical Information Mart for Intensive Care IV) database, which contains comprehensive, deidentified, and well-maintained clinical data on ICU patients, providing an excellent foundation for the development of our predictive model. This study therefore aimed to identify predictors of prolonged LOS in the ICU and attempted to construct a valid predictive model. This could help clinicians and nurses identify patients who require extended ICU stays and develop appropriate interventions to speed up ICU bed turnover and reduce healthcare costs.

## Materials and methods

### Data source and ethics statement

The MIMIC-IV database is available primarily for researchers from the Massachusetts Institute of Technology Computational Physiology Laboratory and Collaborative Research Group (26). This database includes demographic information, vital signs, laboratory indicators, medications, and medical-care information. The database has a large sample, comprehensive information, and long-term patient follow-ups, while being free to use and providing a rich resource for critical-care research (27).

This study adhered to the provisions of the Declaration of Helsinki, and ethics approval was provided by the Ethics Committee and Institutional Review Board of the First Affiliated Hospital of Jinan University. Besides, our authors received permission after completing the “Protecting Human Research Participants” web-based training program from the National Institutes of Health (record ID: 45369280). The requirement for obtaining individual patient consent was waived in the present study since it did not have any direct implications on clinical care, and all confidential health data was anonymized to safeguard patient privacy.

### Study population

**Figure 1:**
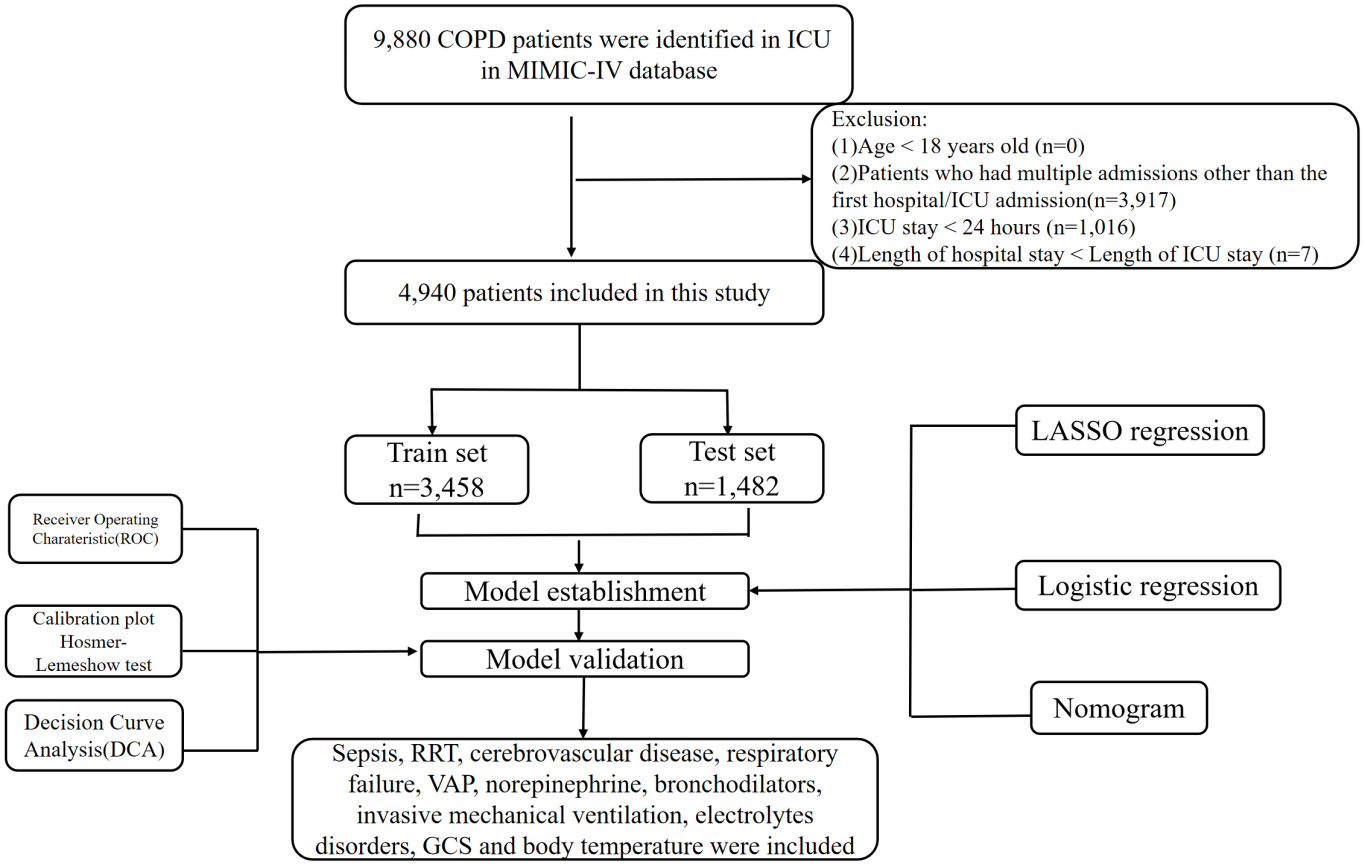
Inclusion and exclusion flowchart of the study.

The number of 69,211 patients admitted to ICU in MIMIC-IV database from 2008–2019. This study used the Ninth Revisions of the International Classification of Diseases (49,120, 49,121, 49,122, 496) and the Tenth Revisions of the International Classification of Diseases (J44, J440, J441, J449) codes to extract 9,980 patients admitted to the ICU with a COPD diagnosis (including acute exacerbated COPD) from the MIMIC-IV database. The following populations were excluded in this study: (1) patients under the age of 18, (2) patients who had multiple admissions other than the first hospital/ICU admission, (3) patients with ICU stays of less than 1 day, including those who died upon ICU admission, and (4) patients with a hospital stay shorter than their ICU stay. The final population for our study was 4,940 patients. Figure 1 illustrates the detailed patient selection process.

### Data extraction

The following data were extracted from the MIMIC-IV database using structured query language (28): (1) general patient data and demographic characteristics: sex, age, weight, height, race, smoking history, marital status, and length of ICU stay; (2) vital signs: body temperature, heart rate, respiratory rate, mean blood pressure, and pulse oximetry-derived oxygen saturation; (3) laboratory test results: pH, glucose, hematocrit, hemoglobin, platelets, white blood cell count, anion gap, bicarbonate, blood urea nitrogen, calcium, chloride, creatinine, sodium, potassium, internal normalized ratio, prothrombin time, partial thromboplastin time(s), PaO_2_, Lactate, total CO_2_, PaCO_2_, lymphocytes, basophils, eosinophils, monocytes, neutrophils and urine output; (4) comorbidities: sepsis, myocardial infarct, congestive heart failure, peripheral vascular disease, cerebrovascular disease, dementia, rheumatic disease, peptic ulcer disease, mild liver disease, uncomplicated diabetes, paraplegia, renal disease, malignant cancer, severe liver disease, metastatic solid tumor, acquired immunodeficiency syndrome, respiratory failure, ventilation associated pneumonia (VAP), hypertension, pneumonia, septic shock, electrolytes disorder and asthma; (5) scores on assessment scales: Charlson comorbidity index, Acute Physiology score III (APSIII), Oxford Acute Severity of Illness score (OASIS), Sequential Organ Failure Assessment (SOFA) score, Glasgow Coma Scale (GCS) score and Braden Scale score; and (6) treatment and medications: renal replacement therapy (RRT), antibiotic, norepinephrine, invasive mechanical ventilation, bronchodilators and glucocorticoids. For vital signs and laboratory test results, we extracted the mean values on the first day of ICU admission. The multiple interpolation (29) was used to process missing data. However, features with a missing rate higher than 20% (height) were removed since we aimed to build a prediction model that can be generalized in the real clinic which should contain accessible data. The missing rate of all extracted variables was shown in Supplementary Figure 1.

### Nomograms construction and validation

After applying the inclusion and exclusion criteria, the final population analyzed comprised 4,940 patients. Given the largeness of the sample, we used the Type 2a scheme TRIPOD (30) (Transparent Reporting of a multivariable prediction model for Individual Prognosis or Diagnosis) for prediction-model building and validation. The data set was first randomly divided into training (*n* = 3,458) and validation (*n* = 1,482) sets at a 7:3 ratio. The predictor variables included in the column line graph (nomogram) were selected in two steps. First, we analyzed the data in the training set using least absolute shrinkage and selection operator (LASSO) regression (31, 32) to select the best risk factors for prolonged ICU stay. LASSO analysis (33, 34) is performed by generating a penalty function that is a compression of the coefficients of the variables in the regression model to prevent overfitting and solve the problem of severe covariance. Second, the most important features selected by the LASSO regression from the training set were used in a multifactorial logistic regression analysis. Variables with *p* < 0.05 were included in the nomogram, and multifactorial analysis was used to predict the respective probabilities of prolonged ICU stay in patients with COPD.

The validation of the prediction model consisted of three main processes: discrimination, calibration, and clinical validity. In this study, the areas under the receiver operating characteristic (ROC) curves (area under curve, AUC) were used to determine the discrimination of the model, the Hosmer-Lemeshow test and calibration plots were used to evaluate its calibration, and decision-curve analysis (DCA) was used to assess its clinical validity.

### Study outcomes

The primary outcome was prolonged ICU length of stay. Detailed ICU admission time and discharge time were recorded for all patients in MIMIC-IV. Length of ICU stay was calculated as the difference between ICU discharge time (icu_outtime) and ICU admission time (icu_intime). Currently, there is no universally accepted definition of prolonged ICU length of stay (35). ICU length of stay >5 days was defined prolonged LOS in the ICU in this study (36) (based on the 75th percentile of ICU LOS in our sample).

### Statistical analysis

We performed a descriptive analysis of the above variables. The baseline data of all patients were grouped by prolonged ICU stay (yes or no). Continuous variables were expressed as medians (25th–75th percentiles); categorical variables were expressed as counts and percentages. The Wilcoxon rank sum test was used to compare group differences for continuous variables, and Chi-squared tests were used to compare categorical variables. For covariance between continuous variables in the logistic regression, we used the variance inflation factor (VIF), with VIF < 4 indicated no multicollinearity among predictors (37).

All data were analyzed and processed using R software (version 4.2.1, https://www.r-project.org/) for statistical analysis and processing (38). Multiple interpolation was performed using the “mice” package, descriptive analysis and comparisons of differences between groups were performed using “tableone” package, LASSO regression analysis was performed using “glmnet” package, multifactor logistic regression analysis was performed using “rms” package, ROC curves were plotted using “pROC” package, and DCA was performed using “rmda” package. A probability value of *p* < 0.05 was considered statistically significant, and all statistical tests were two-sided.

## Results

### Characteristics of included patients

The number of 4,940 patients were included in this study, and 1,255 (25.4%) prolonged ICU stay (length of stay more than 5 days). The baseline characteristics of all patients were lied in Table 1. The median age of all patients was approximately 72 years old, and 2,660 (53.8%) men were included in the study. Compared with the non-prolonged ICU stay patients, prolonged ICU stay patients generally had more comorbidities, medication, treatment, and tended to have more severe illness severity scores (Table 1). The demographics and clinical characteristics of the patients in the training and validation sets are listed in Supplementary Table 1. There was good comparability between the two groups of patients.

**Table 1:** Comparison of baseline data between non-ICU p-LOS and ICU p-LOS cohort

Variables	Total (*n*=4940)	Non-ICU p-LOS Group(*n*=3685)	ICU p-LOS Group(*n*=1255)	*p*-value
**General characteristics**				
Age (years old)	72.00 (64.00, 80.00)	72.00 (64.00, 80.00)	71.00 (63.50, 79.00)	0.005
Sex, male (%)	2660 (53.8)	1970 (53.5)	690 (55.0)	0.368
Weight (kg)	78.80 (65.00, 95.00)	78.40 (64.45, 94.20)	80.00 (65.94, 97.28)	0.007
Race, White (%)	3590 (72.7)	2735 (74.2)	855 (68.1)	<0.001
Smoke (%)	1503 (30.4)	1161 (31.5)	342 (27.3)	0.005
**Vital signs**				
Temperature (°C)	36.80 (36.60, 37.00)	36.80 (36.60, 37.00)	36.90 (36.60, 37.20)	<0.001
Heart rate (beats/minute)	84.00 (75.00, 96.00)	83.00 (74.00, 94.00)	87.00 (75.00, 99.00)	<0.001
Respiratory rate (beats/minute)	19.00 (17.00, 22.00)	19.00 (17.00, 22.00)	20.00 (18.00, 23.00)	<0.001
MBP (mmHg)	76.00 (70.00, 83.00)	76.00 (70.00, 83.00)	75.00 (69.00, 82.00)	0.004
SpO_2_ (%)	96.00 (95.00, 98.00)	96.00 (95.00, 98.00)	96.00 (95.00, 98.00)	0.167
**Laboratory tests**				
pH (units)	7.37 (7.30, 7.42)	7.37 (7.31, 7.42)	7.36 (7.28, 7.42)	<0.001
Glucose (mg/dL)	132.00 (113.00, 161.25)	131.00 (113.00, 159.00)	136.00 (114.00, 167.00)	0.008
Hematocrit (%)	32.50 (28.60, 37.20)	32.50 (28.70, 37.00)	32.40 (28.45, 37.60)	0.743
Hemoglobin (g/dL)	10.60 (9.30, 12.20)	10.60 (9.30, 12.10)	10.50 (9.20, 12.20)	0.604
Platelets (10^9^/L)	196.00 (146.00, 260.00)	196.00 (148.00, 261.00)	195.00 (138.00, 259.00)	0.162
WBC (10^9^/L)	11.40 (8.50, 15.30)	11.10 (8.30, 14.90)	12.10 (8.80, 15.90)	<0.001
Anion gap (mEq/L)	14.00 (12.00, 17.00)	14.00 (12.00, 16.00)	14.00 (12.00, 17.00)	0.011
Bicarbonate (mEq/L)	24.00 (21.50, 27.00)	24.00 (22.00, 27.00)	23.50 (20.50, 27.00)	<0.001
BUN (mg/dL)	22.00 (15.00, 35.00)	21.00 (15.00, 33.00)	25.00 (17.00, 40.00)	<0.001
Calcium (mg/dL)	8.4 (8.00, 8.80)	8.4 (8.00, 8.80)	8.30 (7.80, 8.80)	<0.001
Chloride (mEq/L)	103.00 (99.00, 107.00)	103.00 (99.00, 107.00)	103.00 (99.00, 107.00)	0.436
Creatinine (mg/dL)	1.00 (0.80, 1.50)	1.00 (0.80, 1.50)	1.10 (0.80, 1.80)	<0.001
Sodium (mEq/L)	139.00 (136.00, 141.00)	139.00 (136.00, 141.00)	139.00 (136.00, 142.00)	0.009
Potassium (mEq/L)	4.30 (3.90, 4.70)	4.30 (3.90, 4.70)	4.30 (3.90, 4.80)	0.051
INR	1.30 (1.10, 1.50)	1.30 (1.10, 1.50)	1.30 (1.10, 1.60)	<0.001
PT (s)	13.80 (12.30, 16.30)	13.70 (12.20, 16.10)	14.20 (12.50, 17.40)	<0.001
PTT (s)	31.70 (27.60, 41.20)	31.20 (27.40, 40.10)	33.30 (28.25, 44.45)	<0.001
PaO_2_ (mmHg)	88.00 (60.00, 100.00)	88.00 (60.00, 100.00)	88.00 (60.50, 100.00)	0.764
Lactate (mmol/L)	1.60 (1.10, 2.40)	1.60 (1.10, 2.40)	1.60 (1.10, 2.36)	0.745
Total CO_2_ (mEq/L)	27.00 (23.00, 30.00)	27.00 (24.00, 30.00)	26.00 (23.00, 31.00)	0.049
PaCO_2_ (mmHg)	44.50 (38.00, 53.00)	44.00 (38.00, 53.00)	45.00 (38.00, 55.00)	0.021
Lymphocytes (%)	10.70 (5.80, 17.70)	11.50 (6.10, 18.80)	8.70 (4.80, 14.20)	<0.001
Basophils (%))	0.30 (0.10, 0.50)	0.30 (0.10, 0.50)	0.20 (0.10, 0.40)	<0.001
Eosinophils (%)	0.60 (0.10, 1.70)	0.70 (0.10, 1.90)	0.30 (0.00, 1.20)	<0.001
Monocytes (%)	5.10 (3.20, 7.50)	5.10 (3.30, 7.50)	5.00 (3.00, 7.40)	0.046
Neutrophils (%)	80.40 (71.40, 87.40)	79.70 (70.50, 87.00)	82.80 (74.30, 88.70)	<0.001
Urine output (ml)	1480.00 (950.00, 2260.00)	1530.00 (990.00, 2304.00)	1335.00 (844.50, 2119.50)	<0.001
**Comorbidities**				
Sepsis (%)	2813 (56.9)	1756 (47.7)	1057 (84.2)	<0.001
Myocardial infarct (%)	1150 (23.3)	842 (22.8)	308 (24.5)	0.235
Congestive heart failure (%)	2154 (43.6)	1559 (42.3)	595 (47.4)	0.002
Peripheral vascular disease (%)	959 (19.4)	718 (19.5)	241 (19.2)	0.86
Cerebrovascular disease (%)	745 (15.1)	513 (13.9)	232 (18.5)	<0.001
Dementia (%)	194 (3.9)	152 (4.1)	42 (3.3)	0.254
Rheumatic disease (%)	243 (4.9)	185 (5.0)	58 (4.6)	0.625
Peptic ulcer disease (%)	141 (2.9)	106 (2.9)	35 (2.8)	0.95
Mild liver disease (%)	497 (10.1)	325 (8.8)	172 (13.7)	<0.001
Uncomplicated diabetes (%)	1295 (26.2)	969 (26.3)	326 (26.0)	0.853
Paraplegia (%)	186 (3.8)	118 (3.2)	68 (5.4)	0.001
Renal disease (%)	1229 (24.9)	896 (24.3)	333 (26.5)	0.125
Malignant cancer (%)	763 (15.4)	599 (16.3)	164 (13.1)	0.008
Severe liver disease (%)	164 (3.3)	94 (2.6)	70 (5.6)	<0.001
Metastatic solid tumor (%)	362 (7.3)	287 (7.8)	75 (6.0)	0.039
AIDS (%)	21 (0.4)	15 (0.4)	6 (0.5)	0.934
Respiratory failure (%)	1998 (40.4)	1148 (31.2)	850 (67.7)	<0.001
VAP (%)	180 (3.6)	28 (0.8)	152 (12.1)	<0.001
Hypertension (%)	2165 (43.8)	1638 (44.5)	527 (42.0)	0.138
Pneumonia (%)	935 (18.9)	630 (17.1)	305 (24.3)	<0.001
Septic shock (%)	616 (12.5)	331 (9.0)	285 (22.7)	<0.001
Electrolytes disorders (%)	2210 (44.7)	1417 (38.5)	793 (63.2)	<0.001
Asthma (%)	165 (3.3)	125 (3.4)	40 (3.2)	0.796
**Scores on assessment scales**				
APSIII	45.00 (34.00, 62.00)	42.00 (32.00, 54.00)	62.00 (46.00, 83.00)	<0.001
GCS	14.00 (11.00, 15.00)	14.00 (13.00, 15.00)	11.00 (7.00, 14.00)	<0.001
SOFA	5.00 (3.00, 8.00)	4.00 (2.00, 6.00)	7.00 (5.00, 10.00)	<0.001
Charlson comorbidity index	7.00 (6.00, 9.00)	7.00 (5.00, 9.00)	7.00 (6.00, 9.00)	0.044
Braden score	15.00 (13.00, 16.00)	15.00 (13.00, 17.00)	14.00 (12.00, 16.00)	<0.001
OASIS	33.00 (27.00, 40.00)	31.00 (26.00, 37.00)	39.00 (32.00, 46.00)	<0.001
**Treatment and medications**				
RRT (%)	336 (6.8)	152 (4.1)	184 (14.7)	<0.001
Antibiotic (%)	3684 (74.6)	2534 (68.8)	1150 (91.6)	<0.001
Norepinephrine (%)	1199 (24.3)	608 (16.5)	591 (47.1)	<0.001
Invasive mechanical ventilation (%)	1930 (39.1)	1168 (31.7)	762 (60.7)	<0.001
Bronchodilators (%)	4008 (81.1)	2913 (79.1)	1095 (87.3)	<0.001
Glucocorticoids (%)	2280 (46.2)	1595 (43.3)	685 (54.6)	<0.001

Medians and interquartile ranges (25th and 75th percentiles) were computed for continuous variables, and frequencies and percentages for categorical variables. The Wilcoxon rank-sum test was used to compare group differences for continuous variables, and Chi-square tests for categorical variables. ICU: Intensive Care Units; p-LOS, Prolonged length of stay; RRT, renal replacement therapy; AIDS, Acquired Immune Deficiency Syndrome; VAP, ventilation associated pneumonia; MBP, mean blood pressure; APSIII, acute physiology score III; GCS, Glasgow Coma Score; SOFA, sequential organ failure assessment; OASIS, oxford acute severity of illness score; SpO_2_, pulse oximetry-derived oxygen saturation; PaO_2_: arterial partial pressure of oxygen ; PaCO_2_ : partial pressure of carbon dioxide; WBC, white blood cell count; BUN: blood urea nitrogen; PT: prothrombin time; PTT: partial thromboplastin time; INR: internal normalized ratio.

### Variable filtering of the training set

**Figure 2:**
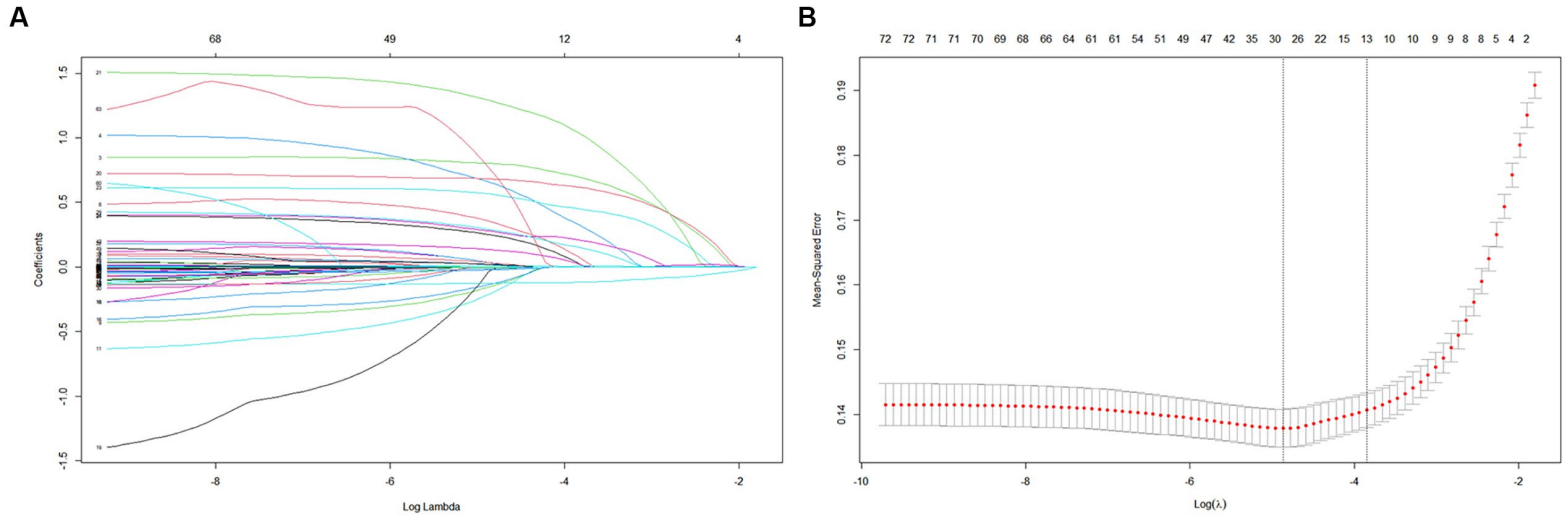
Variable selection by LASSO binary logistic regression model. **(A)** Each curve with different colors represents the change trajectory of each independent variable coefficient, the y-axis is the coefficient value; the upper x-axis is the number of non-zero coefficients in the LASSO model; **(B)** Represented the cross-validation result with different λ value, the left dot line represented lambda.min which was the lowest λ of minimum mean cross-validated error, the right dot line represented the lambda.1se which was the largest value of λ such that error is within 1 standard error of the cross-validated errors for lambda.min.

Among the 72 relevant feature variables extracted, 13 potential predictor variables were selected based on the data from the training set (Figures 2A,B) that had nonzero coefficients in the LASSO regression model. Features fitted to construct prediction models were selected by the largest λ at which the mean square error (MSE) is within one standard error of the minimal MSE (Supplementary Table 2). These predictors were sepsis, renal replacement therapy cerebrovascular disease, respiratory failure, ventilator associated pneumonia norepinephrine, bronchodilators, invasive mechanical ventilation, electrolytes disorders, Glasgow Coma Scale score acute physiology score III oxford acute severity of illness score and body temperature. However, in the multivariate logistic regression, two variables (APSIII and OASIS) were excluded because they were not statistically effects (Supplementary Table 3). Finally, we included 11 variables to construct the model.

### Construction of predictive models

**Figure 3:**
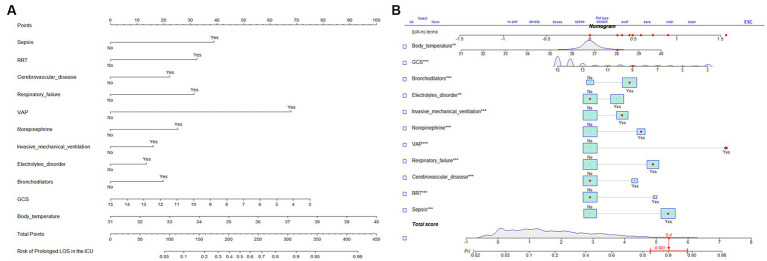
Developed risk nomograms for prolonged ICU length of stay with COPD patients. **(A)** Normal nomograms. **(B)** Dynamic nomograms. p-LOS, prolonged length of stay; RRT, renal replacement therapy; VAP, ventilation associated pneumonia; GCS, Glasgow Coma Score.

The results of the multifactorial logistic regression analysis for sepsis, renal replacement therapy (RRT), cerebrovascular disease, respiratory failure, ventilator associated pneumonia (VAP), norepinephrine, bronchodilators, invasive mechanical ventilation, electrolytes disorders, Glasgow Coma Scale score (GCS) and body temperature were listed in Table 2. All of these predictor variables had significant effects, and hence they were used to construct a nomogram of the risk of prolonged LOS in the ICU for patients with COPD, which is presented in Figure 3A. Each predictor variable indicator corresponds to a set of values on the scale with the score scale on the top (points), the scores of all the indicators were summed to obtain the total score, and the position of the total score on the bottom total points scale (total points) corresponds to the probability of having a prolonged LOS in the ICU for patients with COPD. This study also applied the “shiny app” package of R software,^1^ which is mostly used to help physicians and nurses working in clinical settings to predict risks and individualize patient assessments. As an example to help the reader better understand the nomogram, if a COPD patient with sepsis, respiratory failure, and ventilate associated pneumonia has been treated with bronchodilators, invasive mechanical ventilation, norepinephrine, a Glasgow Coma Score of 9, and a temperature of 38°C during ICU stay. Then the probability of prolonged LOS in his/her ICU was about 90.1% (95%CI, 0.835–0.943; Figure 3B).

**Table 2:** Multivariate logistic regression analysis of the selected significant clinical characteristics in the training set.

Variables	OR^a^	95%CI^b^	*p*-value
GCS	0.86	0.84–0.89	<0.001^*^
Temperature	1.29	1.09–1.53	0.003^*^
**Sepsis**			
Yes	2.46	1.97–3.08	<0.001^*^
No	Reference		
**RRT**			
Yes	2.11	1.51–2.96	<0.001^*^
No	Reference		
**Cerebrovascular disease**			
Yes	1.67	1.30–2.14	<0.001^*^
No	Reference		
**Respiratory failure**			
Yes	2.06	1.69–2.51	<0.001*
No	Reference		
**VAP**			
Yes	4.78	2.86–8.37	<0.001^*^
No	Reference		
**Norepinephrine**			
Yes	1.79	1.46–2.21	<0.001^*^
No	Reference		
**Bronchodilators**			
Yes	1.58	1.21–2.06	<0.001^*^
No	Reference		
**Invasive mechanical ventilation**			
Yes	1.45	1.19–1.75	0.004^*^
No	Reference		
**Electrolytes disorder**			
Yes	1.36	1.12–1.65	0.001^*^
No	Reference		

^*^p < 0.05; ^a^OR odd ratio; ^b^CI confidence interval; GCS, Glasgow Coma Score; RRT, renal replacement therapy; VAP, ventilation associated pneumonia.

### Validation of predictive models

**Figure 4:**
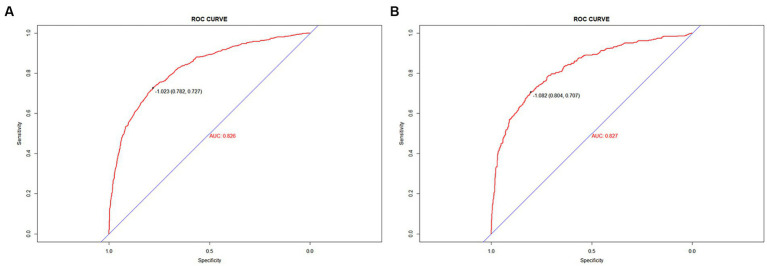
The receiver operating characteristic curve for the train and validation cohort. **(A)** The receiver operating characteristic curve for the train cohort. **(B)** The receiver operating characteristic curve for the validation cohort.

**Figure 5:**
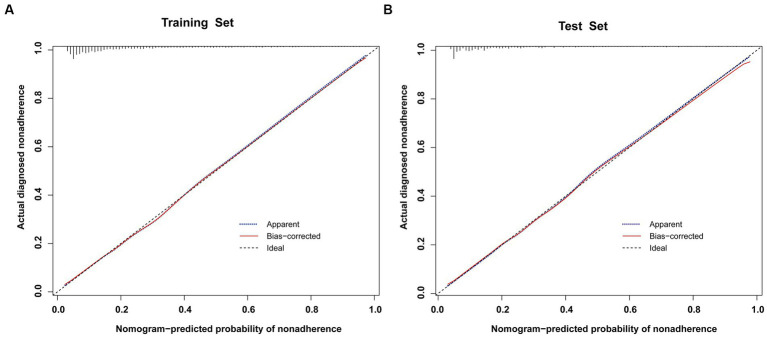
The calibration curve for the train and validation cohort. **(A)** The calibration curve for the train cohort. **(B)** The calibration curve for the validation cohort.

To validate the predictive models, we first performed a VIF test with all variables scoring less than 4. There was no covariance and the model fit was good. The AUC values (equal to C-index) were 0.826 (95%CI, 0.809–0.842) and 0.827 (95%CI, 0.802–0.853) in the training and validation sets, respectively (Figure 4), which indicates good performance. The results of a Hosmer-Lemeshow test indicated strong agreement between the predicted and observed probabilities in the training (χ^2^ = 8.21, *p* = 0.413) and validation (χ^2^ = 8.21, *p* = 0.413) sets. The calibration curves of the nomograms used to predict the risk of prolonged LOS in the ICU for patients with COPD also indicated good consistency (Figures 5A,B). Together these validation results indicate that the nomograms of the model had good predictive effects.

**Figure 6:**
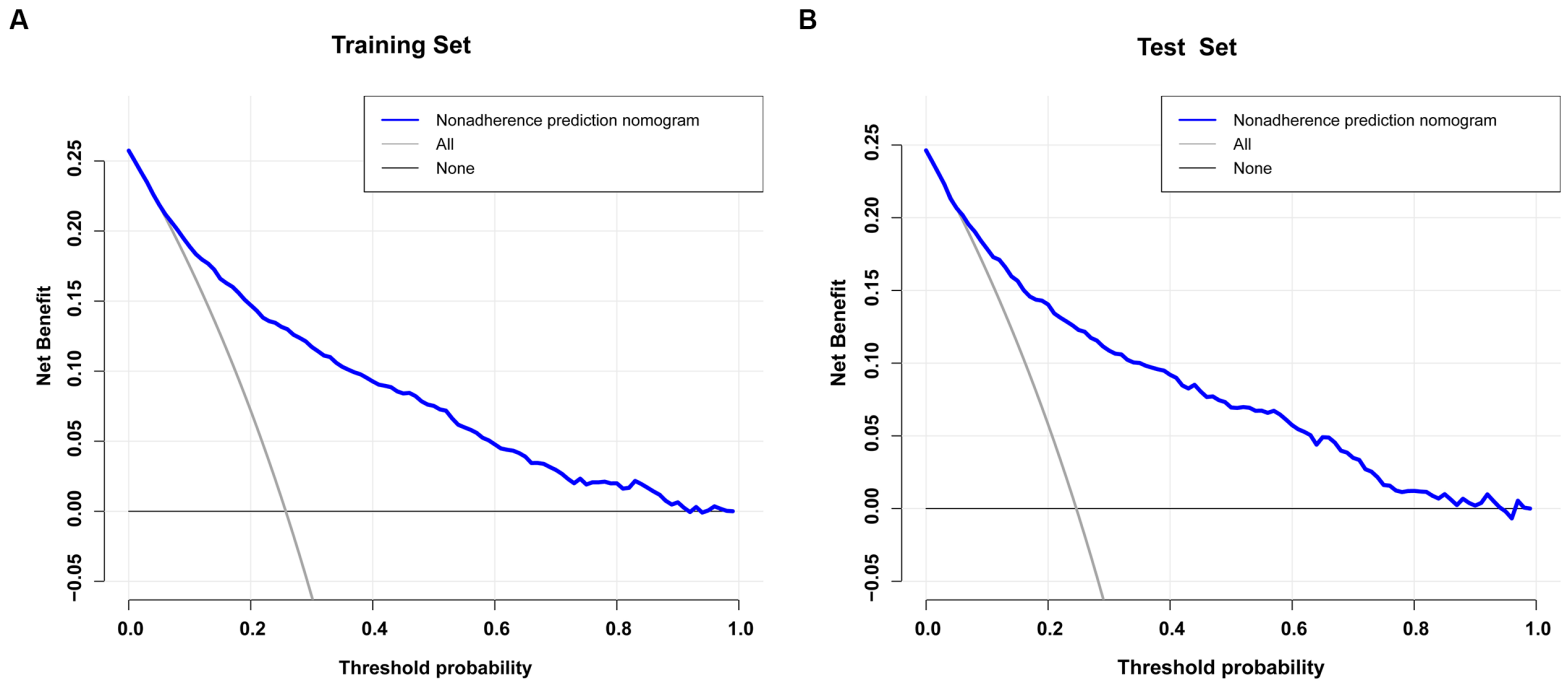
The decision analysis curve for the train and validation cohort. **(A)** The decision analysis curve for the train cohort. **(B)** The decision analysis curve for the validation cohort. The horizontal line means that all samples are negative, and the oblique line means that all samples are positive. The blue line represents the risk nomograms.

The blue line in the DCA graph in Figure 6 indicates the scenarios in which this model predicts the occurrence of prolonged LOS in ICUs for patients with COPD. For comparison purposes, the horizontal and diagonal lines represent the two extreme cases: the former represents all samples being negative, while the latter represents all samples being positive. The DCA results indicated that patients with COPD would obtain higher net clinical benefits when using nomograms to predict prolonged ICU stay in both the training and validation sets (Figures 6A,B). For example, in the validation set, assuming a timely intervention for patients with COPD with a 40% risk of developing an prolonged ICU stay, 9 people would benefit for every 100 interventions.

## Discussion

Column line graphs (nomograms) are simple, reliable, and practical forecasting tools (39). They have been widely used in clinical settings to help predictions and decision-making by identify several relevant predictors (40). We have constructed the first risk prediction model for a prolonged ICU stay in patients with COPD that has good predictive effects, with an AUC of 0.827 (95%CI, 0.802–0.853) in the validation set. The prediction model established in this study was also strongly calibrated and had good clinical validity. Sepsis, RRT cerebrovascular disease, respiratory failure, VAP norepinephrine, bronchodilators, invasive mechanical ventilation, electrolytes disorders, GCS and body temperature of patients with COPD can be used as predictors. We can therefore clinically predict the probability of prolonged ICU stays of patients with COPD by obtaining healthy history information, performing physical examinations, drugs and treatment measures. This prediction model can provide guidance for the development of strategies to prevent prolonged ICU length of stay.

Sepsis is a clinical syndrome that poses a life-threatening risk to patients due to the dysregulation of their response to infections, which leads to organ dysfunction (41). Similar to previous studies, sepsis could result in prolonged ICU stays for patients (42, 43). The treatment of sepsis would take a long time and consume a lot of medical resources, which led to a longer stay in the ICU (44). Renal replacement therapy is a therapeutic approach that utilizes blood purification technology to clear solutes, in order to replace the impaired renal function as well as play a protective and supportive role on organ function (45). The use of RRT will prolong ICU stay in our study. Contrary to our conclusion, a meta-analysis showed that early renal replacement therapy was associated with significantly shorter ICU length of stay (46). This disparity may be explained due to differences in data collection. This study was concerned only with the use of RRT with COPD patients instead of the timing of RRT use.

The GCS is composed of the eye-opening response, verbal response, and body movement (47). It is not surprising that the GCS score is a protective factor for longer LOS in patients with COPD (OR = 0.86, 95% CI = 0.84–0.89, *p* < 0.001), which is consistent with results in clinical practice (48). This is because a lower GCS score is indicative of a more-severe case of impaired consciousness and therefore a longer ICU stay.

Bronchodilators, which include β_2_-adrenergic agonists, anticholinergics, and theophyllines, are the main measures to control symptoms in patients with COPD (49). Bronchodilators were associated with prolonged ICU stay in this study. Because patients with COPD who are treated with bronchodilators usually experience acute exacerbations, they will have increased dyspnea symptoms and declined respiration function and will require ICU observation for a longer duration than nonusers, resulting in a longer ICU stay. Similarly, cerebrovascular disease, one of the major health problems worldwide (50), is accompanied by multiple forms of neurological dysfunction and altered vascular statuses, including stroke. This undoubtedly increases the risk of patient care and prolonged patient stays. In additional, electrolyte disorders are common in the ICU and can seriously affect the blood supply and metabolism (51). This led to longer ICU stays for patients (52).

Previous studies (53–55) have found that the body temperature of a patient on admission affects their LOS in the ICU. Although those studies were performed on other populations, body temperature was still a predictor for the model in this study. Changes in body temperature is one of the important indicators of condition monitoring, and can affect subsequent treatment and prognosis. Geffroy et al. (56) found that patients with early fever were more likely to have a poor prognosis and hence a prolonged ICU stay.

Similar to the results of previous studies (57–61), invasive mechanical ventilation, respiratory failure and ventilator associated pneumonia increased the LOS in the ICU for patients with COPD. This is due to these patients usually had a more-severe gas exchange impairment, require respiratory support, and have a longer ICU stay. However, unlike previous studies (53), norepinephrine was associated with prolonged LOS in the ICU, and was therefore a risk factor. Possible reasons for this are that patients using norepinephrine have unstable circulatory function and greater variability in their condition and LOS, thus requiring more attention and monitoring by nurses and doctors (48).

Our study aimed to construct a nomogram for predicting prolonged LOS among patients with COPD hospitalized in the ICU. This study differs from the previous study in that our outcome of interest is prolonged LOS, while the other study focused on 30-day mortality prediction (62). Furthermore, we utilized a distinct set of variables to identify predictors specifically relevant to extended LOS among COPD patients in the ICU. Our study contributes to the literature by providing healthcare professionals with a practical tool to stratify patients, optimize resource allocation, and improve patient care. By developing a nomogram that can predict prolonged LOS, we offer clinicians an additional means to identify patients at high risk for prolonged ICU LOS, allowing for more informed decision-making and targeted interventions. Overall, our study represents a significant advancement in understanding and managing COPD patients in the ICU, and has important clinical implications for improving patient outcomes and resource utilization.

Identifying patients at risk of prolonged length of stay may help ICU management and avoid ICU a shortage of ICU beds (53). Considering the specificity and complexity of patients with COPD in the intensive care unit, this study selected 4,940 patients with severe COPD from a large intensive care medicine database for a retrospective population-based study. The predictors selected for this study (e.g., temperature, GCS, RRT, and use of invasive mechanical ventilation) are simpler and more accessible to critical care physicians and nurses than respiratory specialty indicators, and help clinicians in their decision making.

### Limitations

This study was based on a large and diverse population of the MIMIC-IV database, but in practice it was subject to several limitations. First, this study had a retrospective single-center design and the sample may be underrepresented. External validation of the line plots was not performed (only internal validation), which may lead to overfitting of the new model, requiring the use of data from other sources to validate the findings. Second, some potentially important factors were not included in our study, making it less comprehensive, including, body mass index (BMI), certain psychosocial factors such as anxiety, depression, and social support. Third, limited by our current capabilities and the extent to which the database was available, we still found that antibiotic and vasopressin use prolonged ICU stays in patients with COPD, which was only expressed simply using categorical variables, which makes the effect of specific dosages on the LOS of patients unclear. Fourth, the included study population meant that important variables such as body temperature ranged from 31°C to 40°C in our nomograms, and there was no way to make good predictions for patients with body temperatures outside this range; this needs to be addressed in future studies. Finally, our research did not specifically examine the repercussions of malnutrition on the intensive care unit length of stay for patients with chronic obstructive pulmonary disease. Malnutrition, a crucial element, has been proven to affect clinical outcomes in individuals with this condition. Regrettably, owing to the absence of exhaustive nutritional data in our patient sample, we could not explore the correlation between malnutrition and length of stay (63). This constraint might have hindered our comprehensive understanding of the intricate factors influencing disease prognosis, potentially leading to an underestimation of nutritional status significance in our conclusions. Consequently, we propose that forthcoming investigations incorporate malnutrition evaluation tools, such as the Mini Nutritional Assessment, Subjective Global Assessment, and Nutritional Risk Screening, to appraise the nutritional condition of patients with chronic obstructive pulmonary disease (64). This approach would yield invaluable insights into malnutrition’s impact on disease progression and outcomes, ultimately aiding in the creation of more focused and efficacious interventions to address this critical aspect of disease management. Moving forward, we intend to encompass as many predictor variables as feasible and validate the model using an external cohort to achieve heightened accuracy in our findings.

## Conclusion

Based on the MIMIC-IV database, we have constructed and validated original predictive and dynamic nomograms for prolonged ICU stays in patients with COPD using 11 easily collected parameters The nomograms (available at https://cht19991225.shinyapps.io/Dynamic_nomogram/) will help medical doctors and nursing practitioners to accurately assess the probability of a prolonged ICU stay in specific COPD patients and to distinguish high-risk patients who may require aggressive medical/nursing measures.

## Data availability statement

The datasets presented in this study can be found in online repositories. The names of the repository/repositories and accession number(s) can be found at: the data were available on the MIMIC-IV website at https://mimic.physionet.org/.

## Ethics statement

The studies involving human participants were reviewed and approved by the Ethics Committee and Institutional Review Board of the First Affiliated Hospital of Jinan University. Written informed consent for participation was not required for this study in accordance with the national legislation and the institutional requirements.

## Author contributions

HC, JLi, and FW created the study protocol, performed the statistical analyses, and wrote the first manuscript draft. JLy and FZ conceived the study, critically revised the manuscript, contributed to data interpretation and manuscript revision. SY and XY assisted with the study design and performed data collection. XH assisted with data collection and manuscript editing. SY confirmed the data and assisted with the statistical analyses. HC maintained the localized database and worked on SQL coding. All authors contributed to the article and approved the submitted version.

## Funding

The work was supported by Guangdong Provincial Key Laboratory of Traditional Chinese Medicine Informatization (2021B1212040007), Clinical Frontier Technology Program of the First Affiliated Hospital of Jinan University, China (no. JNU1AF-CFTP-2022-a01235), and the Science and Technology Projects in Guangzhou, China (nos. 202201020054 and 2023A03J1032).

## Conflict of interest

The authors declare that the research was conducted in the absence of any commercial or financial relationships that could be construed as a potential conflict of interest.

## Publisher’s note

All claims expressed in this article are solely those of the authors and do not necessarily represent those of their affiliated organizations, or those of the publisher, the editors and the reviewers. Any product that may be evaluated in this article, or claim that may be made by its manufacturer, is not guaranteed or endorsed by the publisher.

## Footnotes

^1^ https://cht19991225.shinyapps.io/Dynamic_nomogram/

## Glossary

APSIII: Acute physiology score III
AUC: Area under curve
BMI: Body mass index
BUN: Blood urea nitrogen
COPD: Chronic obstructive pulmonary disease
DBP: Diastolic blood pressure
DCA: Decision curve analysis
GCS: Glasgow coma scale
ICU: Intensive care units
INR: Internal normalized ratio
LASSO: Least absolute shrinkage and selection operator
LOS: Length of stay
MAP: Mean arterial pressure
MIMIC-IV: Medical information mart for intensive care-IV
MSE: Mean square error
OASIS: Oxford acute severity of illness score
PaCO2: Partial pressure of carbon dioxide
PaO2: Arterial partial pressure of oxygen
p-LOS: Prolonged length of stay
PT: Prothrombin time
PTT: Partial thromboplastin time
ROC: Receiver operating characteristic curve
RRT: Renal replacement treatment
SBP: Systolic blood pressure
SOFA: Sequential organ failure assessment
SpO2: Pulse oximetry-derived oxygen saturation
TRIPOD: Transparent reporting of a multivariable prediction model for individual prognosis or diagnosis
VAP: Ventilation associated pneumonia
WBC: White blood cell
