# Supplementary material for: A risk nomogram for predicting prolonged intensive care unit stays in patients with chronic obstructive pulmonary disease
Source: Front Med (Lausanne). 2023 Jul 6;10:1177786. doi: 10.3389/fmed.2023.1177786 (PMC10359115; doi:10.3389/fmed.2023.1177786)
Supplement: Supplementary file 1 [file Data_Sheet_1.docx]

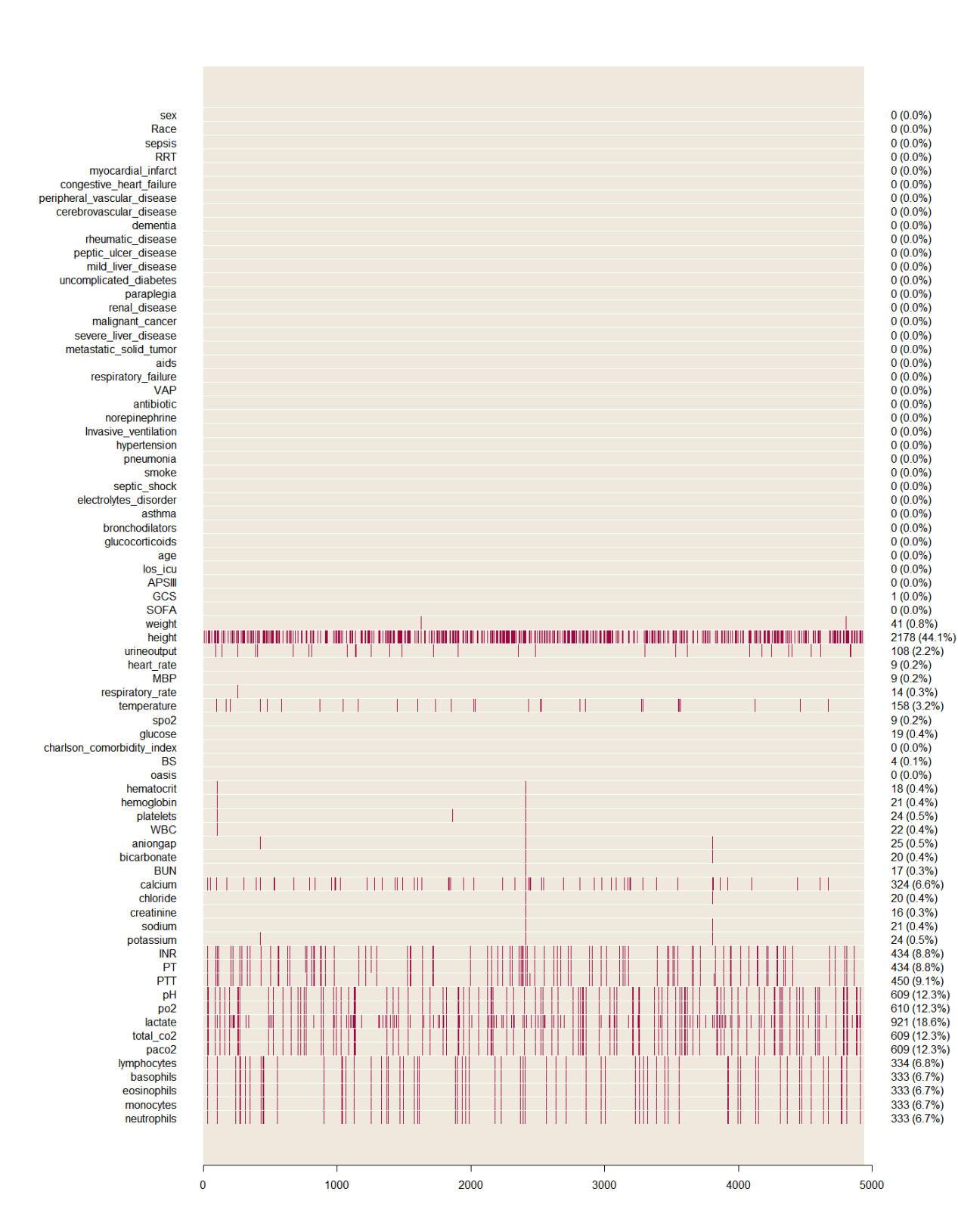


### Supplementary eFigure.1 The missing rate of features across MIMIC-IV databases.

Note: One red block represented one missing value for each feature.

### Supplementary eTable.1 Comparison of baseline data between training and testing cohort

| Variables | Total (n=4940) | Train set (n=3458) | Validation set (n=1482) | P-value |
| --- | --- | --- | --- | --- |
| **General characteristics** | | | | |
| Age (years old) | 72.00 [64.00, 80.00] | 72.00 [64.00, 80.00] | 72.00 [64.00, 80.00] | 0.347 |
| Sex, male (%) | 2660 (53.8) | 1852 (53.6) | 808 (54.5) | 0.554 |
| Weight (kg) | 78.80 [65.00, 95.00] | 78.80 [64.82, 95.60] | 78.80 [65.00, 94.10] | 0.571 |
| Race, White (%) | 3590 (72.7) | 2529 (73.1) | 1061 (71.6) | 0.28 |
| Smoke (%) | 1503 (30.4) | 1055 (30.5) | 448 (30.2) | 0.871 |
| ICU Prolonged length of stay (%) | 1255 (25.4) | 886 (25.6) | 369 (24.9) | 0.618 |
| **Vital signs** | | | | |
| Temperature (°C) | 36.80 [36.60, 37.00] | 36.80 [36.60, 37.00] | 36.80 [36.60, 37.10] | 0.661 |
| Heart rate (beats/minute) | 84.00 [75.00, 96.00] | 84.00 [74.25, 96.00] | 84.00 [75.00, 95.00] | 0.532 |
| Respiratory rate (beats/minute) | 19.00 [17.00, 22.00] | 19.00 [17.00, 22.00] | 19.00 [17.00, 22.00] | 0.395 |
| MBP (mmHg) | 76.00 [70.00, 83.00] | 76.00 [70.00, 83.00] | 76.00 [70.00, 83.00] | 0.554 |
| SpO_2_ (%) | 96.00 [95.00, 98.00] | 96.00 [95.00, 98.00] | 97.00 [95.00, 98.00] | 0.111 |
| **Laboratory tests** | | | | |
| pH (units) | 7.37 [7.30, 7.42] | 7.37 [7.30, 7.42] | 7.37 [7.30, 7.42] | 0.974 |
| Glucose (mg/dL) | 132.00 [113.00, 161.25] | 131.00 [113.00, 160.00] | 133.00 [115.00, 165.00] | 0.041 |
| Hematocrit (%) | 32.50 [28.60, 37.20] | 32.50 [28.60, 37.20] | 32.50 [28.50, 36.80] | 0.317 |
| Hemoglobin (g/dL) | 10.60 [9.30, 12.20] | 10.60 [9.30, 12.20] | 10.60 [9.30, 12.10] | 0.349 |
| Platelets (10^9^/L) | 196.00 [146.00, 260.00] | 197.00 [147.00, 263.00] | 192.00 [144.00, 257.00] | 0.204 |
| WBC (10^9^/L) | 11.40 [8.50, 15.30] | 11.40 [8.40, 15.40] | 11.30 [8.53, 15.00] | 0.407 |
| Anion gap (mEq/L) | 14.00 [12.00, 17.00] | 14.00 [12.00, 17.00] | 14.00 [12.00, 16.00] | 0.723 |
| Bicarbonate (mEq/L) | 24.00 [21.50, 27.00] | 24.00 [21.50, 27.00] | 24.00 [21.50, 27.00] | 0.061 |
| BUN (mg/dL) | 22.00 [15.00, 35.00] | 22.00 [15.00, 35.00] | 22.00 [16.00, 33.00] | 0.934 |
| Calcium (mg/dL) | 8.4 [8.00, 8.80] | 8.40 [8.00, 8.80] | 8.4 [7.90, 8.80] | 0.065 |
| Chloride (mEq/L) | 103.00 [99.00, 107.00] | 103.00 [99.00, 107.00] | 103.00 [100.00, 107.00] | 0.038 |
| Creatinine (mg/dL) | 1.00 [0.80, 1.50] | 1.00 [0.80, 1.60] | 1.00 [0.80, 1.50] | 0.428 |
| Sodium (mEq/L) | 139.00 [136.00, 141.00] | 139.00 [136.00, 141.00] | 139.00 [136.00, 141.00] | 0.759 |
| Potassium (mEq/L) | 4.30 [3.90, 4.70] | 4.30 [4.00, 4.70] | 4.30 [3.90, 4.70] | 0.976 |
| INR | 1.30 [1.10, 1.50] | 1.30 [1.10, 1.50] | 1.30 [1.10, 1.50] | 0.596 |
| PT (s) | 13.80 [12.30, 16.30] | 13.80 [12.20, 16.30] | 13.80 [12.30, 16.30] | 0.414 |
| PTT (s) | 31.70 [27.60, 41.20] | 31.70 [27.60, 41.00] | 31.70 [27.60, 42.00] | 0.968 |
| PaO_2_ (mmHg) | 88.00 [60.00, 100.00] | 88.00 [60.00, 100.00] | 89.00 [60.00, 100.00] | 0.524 |
| Lactate (mmol/L) | 1.60 [1.10, 2.40] | 1.60 [1.10, 2.40] | 1.50 [1.10, 2.38] | 0.305 |
| Total CO_2_ (mEq/L) | 27.00 [23.00, 30.00] | 27.00 [24.00, 31.00] | 26.50 [23.00, 30.00] | 0.15 |
| PaCO_2_ (mmHg) | 44.50 [38.00, 53.00] | 45.00 [38.00, 54.00] | 44.00 [38.00, 53.00] | 0.285 |
| Lymphocytes (%) | 10.70 [5.80, 17.70] | 10.70 [5.80, 17.80] | 10.70 [5.90, 17.50] | 0.76 |
| Basophils (%) | 0.30 [0.10, 0.50] | 0.30 [0.10, 0.50] | 0.30 [0.10, 0.50] | 0.8 |
| Eosinophils (%) | 0.60 [0.10, 1.70] | 0.60 [0.10, 1.70] | 0.60 [0.10, 1.70] | 0.542 |
| Monocytes (%) | 5.10 [3.20, 7.50] | 5.10 [3.30, 7.50] | 5.00 [3.20, 7.50] | 0.772 |
| Neutrophils (%) | 80.40 [71.40, 87.40] | 80.60 [71.40, 87.40] | 80.00 [71.70, 87.60] | 0.859 |
| Urine output (ml) | 1480.00 [950.00, 2260.00] | 1488.50 [944.25, 2262.25] | 1461.00 [957.75, 2250.00] | 0.847 |
| **Comorbidities** | | | | |
| Sepsis (%) | 2813 (56.9) | 1959 (56.7) | 854 (57.6) | 0.547 |
| Myocardial infarct (%) | 1150 (23.3) | 825 (23.9) | 325 (21.9) | 0.152 |
| Congestive heart failure (%) | 2154 (43.6) | 1520 (44.0) | 634 (42.8) | 0.464 |
| Peripheral vascular disease (%) | 959 (19.4) | 653 (18.9) | 306 (20.6) | 0.162 |
| Cerebrovascular disease (%) | 745 (15.1) | 526 (15.2) | 219 (14.8) | 0.729 |
| Dementia (%) | 194 (3.9) | 134 (3.9) | 60 (4.0) | 0.835 |
| Rheumatic disease (%) | 243 (4.9) | 156 (4.5) | 87 (5.9) | 0.051 |
| Peptic ulcer disease (%) | 141 (2.9) | 103 (3.0) | 38 (2.6) | 0.479 |
| Mild liver disease (%) | 497 (10.1) | 331 (9.6) | 166 (11.2) | 0.091 |
| Uncomplicated diabetes (%) | 1295 (26.2) | 919 (26.6) | 376 (25.4) | 0.397 |
| Paraplegia (%) | 186 (3.8) | 115 (3.3) | 71 (4.8) | 0.017 |
| Renal disease (%) | 1229 (24.9) | 856 (24.8) | 373 (25.2) | 0.785 |
| Malignant cancer (%) | 763 (15.4) | 529 (15.3) | 234 (15.8) | 0.693 |
| Severe liver disease (%) | 164 (3.3) | 117 (3.4) | 47 (3.2) | 0.768 |
| Metastatic solid tumor (%) | 362 (7.3) | 245 (7.1) | 117 (7.9) | 0.347 |
| AIDS (%) | 21 (0.4) | 17 (0.5) | 4 (0.3) | 0.39 |
| Respiratory failure (%) | 1998 (40.4) | 1401 (40.5) | 597 (40.3) | 0.904 |
| VAP (%) | 180 (3.6) | 118 (3.4) | 62 (4.2) | 0.214 |
| Hypertension (%) | 2165 (43.8) | 1526 (44.1) | 639 (43.1) | 0.531 |
| Pneumonia (%) | 935 (18.9) | 650 (18.8) | 285 (19.2) | 0.751 |
| Septic shock (%) | 616 (12.5) | 428 (12.4) | 188 (12.7) | 0.8 |
| Electrolytes disorder (%) | 2210 (44.7) | 1553 (44.9) | 657 (44.3) | 0.731 |
| Asthma (%) | 165 (3.3) | 111 (3.2) | 54 (3.6) | 0.489 |
| scores on assessment scales | | | | |
| APSIII | 45.00 [34.00, 62.00] | 45.00 [34.00, 61.75] | 46.00 [34.00, 62.00] | 0.374 |
| GCS | 14.00 [11.00, 15.00] | 14.00 [11.00, 15.00] | 14.00 [11.00, 15.00] | 0.32 |
| SOFA | 5.00 [3.00, 8.00] | 5.00 [3.00, 7.00] | 5.00 [3.00, 8.00] | 0.5 |
| Charlson comorbidity index | 7.00 [6.00, 9.00] | 7.00 [6.00, 9.00] | 7.00 [5.25, 9.00] | 0.48 |
| Braden score | 15.00 [13.00, 16.00] | 15.00 [13.00, 17.00] | 15.00 [13.00, 16.00] | 0.073 |
| OASIS | 33.00 [27.00, 40.00] | 33.00 [27.00, 39.00] | 33.00 [27.00, 40.00] | 0.87 |
| **Treatment and medications** | | | | |
| RRT (%) | 336 (6.8) | 236 (6.8) | 100 (6.7) | 0.97 |
| Antibiotic (%) | 3684 (74.6) | 2565 (74.2) | 1119 (75.5) | 0.343 |
| Norepinephrine (%) | 1199 (24.3) | 817 (23.6) | 382 (25.8) | 0.114 |
| Invasive mechanical ventilation (%) | 1930 (39.1) | 1352 (39.1) | 578 (39.0) | 0.975 |
| Bronchodilators (%) | 4008 (81.1) | 2809 (81.2) | 1199 (80.9) | 0.818 |
| Glucocorticoids (%) | 2280 (46.2) | 1577 (45.6) | 703 (47.4) | 0.249 |

Note: Medians and interquartile ranges (25th and 75th percentiles) were computed for continuous variables, and frequencies and percentages for categorical variables. The Wilcoxon rank-sum test was used to compare group differences for continuous variables, and Chi-square tests for categorical variables.

Abbreviations: ICU: Intensive Care Units; RRT, renal replacement therapy; AIDS, Acquired Immune Deficiency Syndrome; VAP, ventilation associated pneumonia; MBP, mean blood pressure; APSIII, acute physiology score III; GCS, Glasgow Coma Score; SOFA, sequential organ failure assessment; OASIS, oxford acute severity of illness score; SpO_2_, pulse oximetry-derived oxygen saturation; PaO_2_: arterial partial pressure of oxygen ; PaCO_2_ : partial pressure of carbon dioxide WBC, white blood cell count; BUN: blood urea nitrogen; PT: prothrombin time; PTT: partial thromboplastin time; INR: internal normalized ratio.

### Supplementary eTable.2 Coefficients of LASSO repression

| Feature | coefficient |
| --- | --- |
| sepsis | 0.0943259992 |
| RRT | 0.0688507908 |
| cerebrovascular_disease | 0.0118332104 |
| respiratory_failure | 0.1078343523 |
| VAP | 0.2343109520 |
| norepinephrine | 0.0938897659 |
| Invasive_mechanical_ventilation | 0.0387046714 |
| electrolytes_disorder | 0.0231498836 |
| bronchodilators | 0.0023176870 |
| APSIII | 0.0001430947 |
| GCS | -0.0234623712 |
| temperature | 0.0028512612 |
| oasis | 0.0008488503 |

Note: Features fitted to construct prediction models were selected by the largest λ at which the mean square error (MSE) is within one standard error of the minimal MSE.

Supplementary eTable.3 Multivariate logistic regression analysis of the selected clinical characteristics in the training set according to LASSO repression

| Variables | OR^a^ | 95%CI^b^ | p-value |
| --- | --- | --- | --- |
| GCS  Temperature  APSIII  OASIS | 0.87  1.29  0.99  1.07 | 0.84-0.90  1.08-1.53  0.99-1.01  0.92-1.02 | <0.001*  0.004*  0.700  0.317 |
| Sepsis  Yes  No | 2.44  Reference | 1.95-3.06 | <0.001* |
| RRT  Yes  No | 2.12  Reference | 1.50-3.00 | <0.001* |
| Cerebrovascular disease  Yes  No | 1.67  Reference | 1.30-2.14 | <0.001* |
| Respiratory failure  Yes  No | 2.03  Reference | 1.67-2.49 | <0.001* |
| VAP  Yes  No | 4.82  Reference | 2.88-8.45 | <0.001* |
| Norepinephrine  Yes  No | 1.79  Reference | 1.44-2.22 | <0.001* |
| Bronchodilators  Yes  No | 1.57  Reference | 1.48-4.23 | <0.001* |
| invasive mechanical ventilation  Yes  No | 1.38  Reference | 1.11-1.71 | 0.004* |
| Electrolytes disorder  Yes  No | 1.37  Reference | 1.13-1.66 | 0.001 * |

Notes: *P< 0.05；

Abbreviations: ^a^OR, odds ratio; ^b^CI, confidence interval; GCS, Glasgow Coma Score; APSIII, acute physiology score III; OASIS, oxford acute severity of illness score; RRT, renal replacement therapy; VAP, ventilation associated pneumonia.
